# Towards promoting timely treatment: Uncovering the determinants of prompt malaria care seeking behavior among febrile children under-five years in Tanzania

**DOI:** 10.1371/journal.pone.0319913

**Published:** 2025-04-10

**Authors:** Huda Omary, Suleiman Chombo, Pankras Luoga, Jackline Vicent Mbishi, Heavenlight A. Paulo, John Andrew, Abdallah Zacharia, Isaac Y. Addo

**Affiliations:** 1 Department of Parasitology and Medical Entomology, Muhimbili University of Health and Allied Sciences, Dar es Salaam, Tanzania; 2 Department of Epidemiology and Biostatistics, Muhimbili University of Health and Allied Sciences, Dar es Salaam, Tanzania; 3 Department of Development Studies, Muhimbili University of Health and Allied Sciences, Dar es Salaam, Tanzania; 4 University of Galway, Galway, Ireland; 5 General Practice Clinical School, Faculty of Medicine and Health, The University of Sydney, Sydney, Australia; 6 Centre for Social Research in Health, University of New South Wales, Sydney, Australia; King Caesar University, UGANDA

## Abstract

**Background:**

Prompt diagnosis and effective treatment within 24 hours of fever onset is crucial for reducing malaria-related morbidity and mortality in under five children. However, research on the prompt care-seeking behaviors and their determinants in this demographic is limited. This study examined the prevalence of prompt care-seeking behaviors among under five febrile children in Tanzania and the associated determinants.

**Methods:**

This cross-sectional study analyzed data from the 2022 Tanzania Demographic and Health Survey (TDHS), including a nationally representative and weighted sample of 1,050 under-five children who experienced fever within two weeks prior to the survey. A weighted univariable and multivariable modified Poisson regression model with robust estimator was used to examine the association between prompt care seeking behaviors and explanatory variables, including child and caregivers’ factors.

**Results:**

The prevalence of prompt care seeking for febrile children was 43.2%. Caregivers of female children had 18% less prevalences of seeking prompt care (95% CI 0.68–0.98) compared to caregivers with their male children. Caregivers aged 25–34 and 35–49 years had 36% (95% CI 1.05–1.78) and 61% (95% CI 1.16–2.23) higher prevalences of seeking prompt care for their children respectively, compared to younger caregivers aged 15–24 years. Caregivers with at least primary education had 50% (95% CI 1.12–2.02) higher prevalences of seeking prompt care compared to those with no formal education. Additionally, an increase in one household member was associated with a 3% increase in the prevalence of seeking prompt malaria care (95% CI 1.01–1.05).

**Conclusion:**

In Tanzania, female children under five experience delays in care-seeking for malaria, whereas older and more educated caregivers are more likely to seek timely treatment for their children. To enhance health outcomes in this vulnerable group, targeted interventions should prioritize raising awareness among caregivers, particularly younger ones and prompting equitable care seeking to all children regardless of sex.

## Background

Malaria is a preventable and curable yet life-threatening transmitted through the bite of mosquitoes infected with the protozoan parasite Plasmodium [1]. Of the five parasite species that infect humans, *Plasmodium falciparum* and *Plasmodium vivax* poses the greatest threat [2,3]. The World Health Organization (WHO), reported that malaria caused 249 million cases globally in 2022, with 93.6% of the cases occurred in the WHO African Region. That same year, malaria accounted for 608,000 deaths, and 94.5% of which were from the WHO African Region where under five children accounted for 78.1% of the deaths [4].

In 2022, Tanzania, an East African country alone accounted for 4% of global malaria deaths, making it one of the four African countries responsible for just over half (52%) of all malaria deaths [4]. The entire country is considered at risk of malaria although the prevalence varies geographically with highest rate at 23% and the lowest at < 1% [5]. *Plasmodium falciparum* is responsible for 96% of all malaria infection in the country while *P. vivax* and *P. ovale* account for the remaining 4% [6]. To date, malaria remains a significant public health issue in the country, particularly among vulnerable populations such as children and pregnant women. It contributes to more than 10% of deaths among under five children with a prevalence rate of 7% in this vulnerable cohort making them disproportionately affected by the disease [7]. This vulnerability is primarily attributable to the absence of acquired maternal immunity and the immaturity of their immune systems, leaving them susceptible to the severe forms of the disease [8].

Most malaria deaths occur at home without appropriate medical care, and when care is sought, it is often too late [9]. When prevention fails, effective malaria case management with first-line Artemisinin Combination Therapy (ACT) is crucial for preventing complications and mortality in children under five [10]. The WHO emphasizes that early diagnosis and prompt treatment, within 24 hours of symptom onset, are essential for successful malaria control [9,11–13]. To support this, in April 2012, WHO’s Global Malaria Control Program launched the “3 Ts” initiative (Test, Treat, and Track) aimed at enhancing malaria testing, ensuring drug administration aligns with test results, and improving case reporting. This initiative mandates that malaria cases be tested and treated based on results from Rapid Diagnostic Tests (RDTs) or microscopy, hence promoting responsible health-seeking behavior when individuals suspect they have malaria symptoms [14].

Malaria’s early symptoms can resemble those of other febrile conditions, so individuals experiencing fever should seek care at a suitable healthcare facility to rule out malaria or other potential causes [14]. However, in sub-Saharan Africa, high malaria-related mortality rates particularly among under five suggest that only a small proportion of malaria cases receive timely treatment [13]. The risk of death from severe malaria is highest within the first 24 hours, but many patients in endemic settings seem to live far from healthcare facilities, making timely treatment difficult [15]. A systematic review in Kenya reported that only 5% of fever cases received prompt treatment with an anti-malarial drug [16]. Another study conducted in children of 15 years and under in Equatorial Guinea documented that close to half of the children (46.7%) received treatment at least 24 hours after the onset of the symptoms with the median delay in seeking care being 2.8 days within a range of 2–15 days [9]. These findings highlight substantial delays in seeking treatment for malaria symptoms among children in several African countries and emphasize the need for targeted studies and interventions to improve timely access to malaria care.

Despite Tanzania’s high malaria prevalence and mortality rates, studies on prompt malaria care-seeking behavior in the country remain scarce. A study conducted in Dodoma, the capital city of Tanzania showed that only 55.4% of under five children received care within 24 hours of fever onset [11]. However, to the best of our knowledge, no extensive, nationwide studies have been conducted to examine malaria care-seeking behavior among caregivers of febrile children. This gap highlights a critical need for research to better understand regional differences in care-seeking behavior and their implications for malaria outcomes.

This present study is significant as early diagnosis and treatment of malaria are essential for reducing its severe health impacts on young populations [17]. Understanding the determinants of prompt diagnosis and timely treatment of malaria in young children can help inform policy makers and health promoters to reduce malaria-related morbidity and mortality in the country. Thus, the study has the potential to shape malaria policy and prevention strategies in Tanzania, contributing to the achievement of the Sustainable Development Goal (SDG) 3 and Target 3.3. which aims to ensure healthy lives and well-being for all ages by ending epidemics like AIDS, tuberculosis, and malaria [17]. This study, therefore, aims to investigate the prevalence and determinants of prompt malaria care-seeking behavior in this demographic within Tanzania.

## Methods

### Study design and population

The study utilized an analytical cross-sectional design based on secondary data obtained from the 2022 Tanzania Demographic Health Survey (TDHS). We used the DHS dataset for this study due to its comprehensive, nationally representative coverage of all regions and districts in Tanzania, making it uniquely suited for addressing the research objectives. Conducting a similar study using primary data would present significant challenges, including the extensive financial costs of nationwide data collection, the logistical complexities of accessing remote areas, the substantial human resources required for survey administration, and the prolonged time frame needed to achieve comparable coverage. These constraints make primary data collection impractical for a study of this scale, whereas the DHS dataset provides a reliable, efficient, and cost-effective alternative.

The study population consisted of under five children who had experienced fever in the past two weeks prior to the survey, and their caregivers as reported in the 2022 TDHS. A total weighted sample of 1050 caregivers with under-five children were included in this study. In this study, the term “caregivers” refers specifically to mothers, as indicated by the data available. It is important to clarify that this terminology does not imply or reinforce any gender-based assumptions about caregiving roles. We are simply utilizing the data as it was provided, without making any claims about the gender of caregivers. The focus is on the available information, and we acknowledge that caregivers can be of any gender.

### Study variables

#### Outcome variable.

The outcome variable for this study was defined as “whether children with fever whose advice or treatment (care) were sought same or next day”. The outcome variable was defined as binary variable, with value of 1 “yes” implying those children with fever whose care was sought on the same or next day, otherwise, the value was defined as 0 “no”.

#### Independent variables.

The selection of the variables included in this study was based on literature [14,18,19]. The variables were based on child’s characteristics (child’s age and sex), maternal characteristics (mother’s age, education, marital status and parity), household characteristics (residence, wealth index, sex of head of household, household size, bed-net usage and zone) as well as health system characteristics (time to nearest health facility and health insurance). All variables were used as categorical with different defined levels (see Table 1) in exception of household size which was used as continuous variable.

**Table 1:** Social demographic characteristics of the respondents (N = 1050).

Variables	Number (%)
**Malaria prompt care seeking**	
Yes	453 (43.2)
No	597 (56.8)
**Childs’ characteristics**	
Age	
24 months or less	481 (45.8)
Greater than 24 months	569 (54.2)
**Sex**	
Male	551 (52.5)
Female	499 (47.5)
**Caregivers’ characteristics**	
Age	
15–24 years	265 (25.2)
25–34 years	507 (48.3)
35 + years	278 (26.5)
Education	
No education	182 (17.4)
Primary	602 (57.3)
Secondary or higher	266 (25.3)
Parity	
1	167 (15.9)
2–4	605 (57.6)
5 +	278 (26.5)
Residence	
Urban	314 (29.9)
Rural	736 (70.1)
Marital status	
Never in married	70 (6.6)
Married/co-habiting	842 (80.2)
Others	138 (13.2)
**Household characteristics**	
Wealth index	
Poor	366 (34.8)
Middle	188 (17.9)
Rich	496 (47.3)
Sex of head of household	
Male	769 (73.2)
Female	281 (26.8)
Household member size (median) IQR	6 (4–8)
Zone	
Western/lake	498 (47.4)
Northern	169 (16.1)
Central	68 (6.5)
Southern	77 (7.3)
Eastern/Zanzibar	238 (22.7)
Usage of bed-nets (n = 1035)	
No	263 (25.4)
Yes	773 (74.6)
**Health system characteristics**	
Health insurance	
No	978 (93.1)
Yes	72 (6.9)
Time to nearest facility	
30 minutes or less	751 (71.5)
More than 30 minutes	299 (28.5)

### Sampling techniques

The DHS utilizes a two-stage cluster sampling procedure. In the first stage, the country was divided into regions, which were further stratified into urban and rural areas to ensure adequate representation of both populations. Primary sampling units (PSUs), typically census enumeration areas (EAs) or “clusters” were selected from districts of Tanzania using probability proportional to size (PPS), ensuring that larger EAs had a higher chance of being selected and achieving a representative population distribution. In the second stage, households were systematically selected from the available list of all households within the selected clusters. A total of 394 PSUs (clusters) and 965 households were included in the study. From the selected household, all women aged 15-49 and with under five child who had fever in last two weeks, found on her usual residents or slept in the household the night before the survey were eligible to be interviewed. The detailed procedure for the survey is descripted in the DHS final report [5].

### Data management and analysis

Data were checked for missing values. All participants with missing values on the outcome variable of interest were dropped from the study. A continuous variable, time to nearest facility was changed into categorical variable with two categories (30 minutes or less and more than 30 minutes). This was done to ensure an easy comparison between participants living further from the health facilities and those living nearby the health facilities. Some of the categorical variables with many categories such a marital status, level of education, wealth index and zone (region) were re-categorized into fewer categories to simplify the analysis and enhance the interpretability of the results.

The variable selection criteria used in this study was based on previous literatures [14,18,19], important confounders and external knowledge on malaria seeking behavior. The selection of variables for inclusion into multivariable model was not based on corresponding p-values from the univariable analyses. Therefore, all variables included in univariable model were taken into multivariable model. The study used weighted univariable and multivariable modified Poisson regression model with robust estimator. To account for weighted analysis for DHS dataset, weight variable was created from the variable v005 (weight = v005/1,000,000). Then all data were set to survey using the Stata command*, svyset [pweight=weight], psu(v021) strata(v023). Then, svy* command in Stata was used to perform the weighted analyses. The weighted Modified Poisson regression was opted instead of unweighted because the data collection involved the complex sampling design. Hence, weighting was meant to ensure accurate and representative estimates are obtained.

The modified Poisson regression was used because the outcome variable was a common event (had prevalence > 10%) [20,21]. The robust method for standard error estimation was opted to account for overdispersion which is commonly observed in binary data, hence providing more accurate and reliable standard errors. The analysis was done using Stata 17 software. The independent variable in the model was termed significant if had a p-value less than 0.05.

### Ethical considerations

Ethical approval was not required for this study, as our analysis utilized publicly available data. However, the DHS reports that both written and verbal informed consent were obtained from all participants. Before the survey began, ethical clearance was obtained, and all ethical guidelines governing the use of human participants were strictly followed. The methods were conducted in accordance with the relevant guidelines and regulations, including the Declaration of Helsinki.

## Results

### Respondent background characteristics

The results indicate that 453 caregivers (43.2%) sought prompt malaria care either on the same day or the following day after their children under five years of age developed a fever. The sampled participants had a median household size of 6 members per household with an inter-quartile range of 4 household members. Children aged 24 months or less were 481 (45.8%) and female children were 499 (47.5%). Of all caregivers, 265 (25.2%) had age ranging from 15-24 years, 507 (48.3%) from 25-34 years and 278 (26.5%) had age of 35 years and above.

Regarding education background, 182 (17.4%) of caregivers had no formal education. Of all participants, 167 (15.9%) reported having a single child and 842 (80.2%) were married/Co-habiting and 70 (6.6%) were never married. Households with female heads were 281 (26.8%). Of all 1050 participants, only 72 (6.9%) reported having access to health insurance. Likewise, 299 (28.5%) reported living more than 30 minutes to the nearest health facility (Table 1).

### Modified multivariable Poisson regression model with robust estimator

Based on the findings, after controlling for confounders, a mothers’ age, level of education, childs’ sex, and household size were found to be significant factors associated with prompt care-seeking for children at a 95% confidence level. Caregivers aged 25–34 years had a 36% higher prevalence of seeking care for their child on the same day or the next day compared to caregivers under 25 years (95% CI: 1.37 (1.05, 1.78)). Caregivers aged 35–49 years had a 61% higher prevalence of timely care-seeking for their under-five children compared to caregivers under 25 years (95% CI: 1.61 (1.16, 2.23)).

Having a female child was associated with an 18% less prevalences of a mother seeking prompt malaria care compared to having a male child (95% CI: 0.82 (0.68, 0.98)). Caregivers with primary education had 50% higher prevalence of seeking timely malaria care for their children compared to caregivers with no formal education (95% CI: 1.50 (1.12, 2.02)). Additionally, caregivers with secondary or higher education had 36% higher prevalence of seeking malaria prompt care as compared to caregivers with no formal education, although this result was not statistically significant at the 95% confidence interval (95% CI: 1.36 (0.94, 1.95)). As one member increase in household size, the prevalence of a mother seeking malaria prompt care for her child will increase by 3%. (95% CI: 1.03 (1.01, 1.05)).

The usage of bed nets and access to health insurance were associated with a 4% (95% CI: 1.04 (0.85,1.27)) and 7% (95% CI: 1.07 (0.76, 1.51)) higher prevalences of caregivers seeking prompt malaria care for their children, respectively, compared to those without bed nets or health insurance. Caregivers with two to four children had 17% less prevalences of seeking malaria prompt care compared to caregivers with one child (95% CI: 0.83 (0.62, 1.10)). Caregivers with five or more children had 27% less prevalences of seeking prompt malaria care compared to caregivers with one child (95% CI: 0.73 (0.50, 1.05)). But, bed-net usage as well as health insurance and parity were not significant for this study (Table 2).

**Table 2:** Factors associated with malaria prompt care seeking behavior on same or next day.

Variables	Univariable model		Multivariable model	
	Crude prevalence ratio	*P*-value	Adjusted prevalence ratio	*P*-value
**Child’s age**				
24 months or less	1		1	
Greater than 24 months	1.01 (0.84, 1.22)	.922	0.98 (0.82, 1.18)	.83
**Sex of child**				
Male	1		1	
Female	0.79 (0.65, 0.96)	.015**	0.82 (0.68, 0.98)	.031**
**Age of a caregiver (years)**				
15-24	1		1	
25-34	1.32 (1.04, 1.67)	.024**	1.36 (1.05, 1.78)	.022**
35+	1.43 (1.11, 1.85)	.006**	1.61 (1.16, 2.23)	.004**
**Education of a caregiver**				
No formal education	1		1	
Primary	1.52 (1.11, 2.07)	.009**	1.50 (1.12, 2.02)	.007**
Secondary or higher	1.49 (1.06, 2.08)	.021**	1.36 (0.94, 1.95)	.104
**Parity**				
First child	1		1	
2 to 4	1.04 (0.81, 1.34)	.745	0.83 (0.62, 1.10)	.185
5+	1.04 (0.78, 1.38)	.778	0.73 (0.50, 1.05)	.092
**Residence**				
Urban	1		1	
Rural	0.77 (0.64, 0.93)	.006**	0.87 (0.68, 1.10)	.236
**Marital status**				
Never in married	1		1	
Married/Co-habiting	1.59 (0.96, 2.65)		1.39 (0.83, 2.32)	.212
Others	1.45 (0.82, 2.56)	.073	1.37 (0.79, 2.37)	.263
**Wealth index**				
Poor	1		1	
Middle	0.97 (0.74, 1.26)	.812	0.92 (0.70, 1.21)	.571
Rich	1.29 (1.06, 1.58)	.013**	1.13 (0.86, 1.49)	.382
**Sex of head of household**				
Male	1		1	
Female	0.87 (0.68, 1.10)	.242	0.88 (0.67, 1.14)	.33
**Household member size**	1.02 (1.0, 1.04)	.08	1.03 (1.01, 1.05)	.010**
**Zone**				
Western/Lake	1			
Northern	0.86 (0.65, 1.12)	.26		
Central	0.87 (0.57, 1.35)	.54		
Southern/Southern highlands	1.03 (0.77, 1.38)	.832	dropped	
Eastern/Zanzibar	1.15 (0.92, 1.42)	.222		
**Usage of bed-nets**				
No	1		1	
Yes	1.15 (0.93, 1.43)	.198	1.04 (0.85, 1.27)	.735
**Health insurance**				
No	1		1	
Yes	1.20 (0.86, 1.66)	.28	1.07 (0.76, 1.51)	.691
**Time to nearest facility**				
30 minutes or less	1		1	
More than 30 minutes	0.90 (0.74, 1.10)	.294	1.04 (0.84, 1.29)	.699

## Discussion

This study investigated the prevalence of prompt malaria care-seeking behavior among children under five in Tanzania and its associated factors. The findings revealed that 43.2% of children received prompt malaria care either on the same day or the following day, while the remaining children did not receive timely care. This is concerning, given malaria’s status as a leading cause of fever among children in Tanzania, where timely care is essential for reducing the disease’s morbidity and mortality. The observed prevalence is lower than the 100% rate reported in a previous study using data from the 2010 Tanzania Demographic and Health Survey (DHS) [22], and is comparable to the 44.6% reported in a cross-sectional study conducted in Dodoma, Tanzania [11]. Our prevalence rate is also similar to the 46.3% reported in a study conducted in neighbouring country, Malawi [23], indicating that these trends may be regionally consistent. Importantly, our study utilized the most recent DHS data (2022), which reflects the latest trends and changes in health-seeking behavior. This dataset benefits from updated methodologies, and broader coverage, offering a more accurate representation of the current situation. Additionally, the COVID-19 pandemic may have influenced health-seeking behavior, potentially altering caregivers’ concerns about visiting healthcare facilities or shifting health priorities.

Findings from this study show that both childhood and caregivers’ factors, are associated with the prevalence of prompt malaria care seeking behavior among febrile children under-five years in Tanzania. In terms of child-related factors, sex was significantly associated with the prevalence of prompt malaria care seeking behavior among febrile children. Consistent with other studies [15,24], this study found that being classified as male or female significantly influenced a mother’s likelihood of seeking prompt malaria care. Specifically, having a female child was associated with an 18.3% less likelihood of a mother seeking malaria care on the same or next day compared to having a male child. This is probably because male children may be perceived as future breadwinners or bearers of the family name, leading to quicker and more decisive healthcare actions when they fall ill [25,26].

Also, parents are more willing to invest in healthcare for male children, perceiving them as having greater economic value in future than female children [27]. This underscores the need for targeted public health campaigns that directly address cultural biases favoring male children. These campaigns should focus on raising awareness about the importance of providing equal access to timely healthcare for both male and female children. Additionally, there is a critical need for gender-sensitive education that challenges existing gender norms and highlights that prompt medical care is equally crucial for all children, regardless of sex. By emphasizing the equal value of both male and female children in the context of healthcare, such initiatives can help reduce gender-based disparities and improve health outcomes for all children. Concerning caregivers’ factors, age and education level were significantly associated with prompt malaria care seeking behavior for under five children in Tanzania. Our study found that children whose caregivers were 25 years and older were more likely to seek prompt malaria care compared to those with younger caregivers. This may be due to the greater experience and maturity of older caregivers, leading to better health literacy and understanding of the importance of timely treatment. Additionally, older caregivers may have more stable socioeconomic conditions and better access to resources, facilitating quicker access to healthcare services. The same results have been in Malawi and Uganda [23,28]

Another interesting finding was that caregivers that had attained primary and secondary education were more likely to seek prompt healthcare for their febrile children than those with no formal educational background. Although the association is not significant for individuals with secondary education, these findings underscore the critical role of maternal education in malaria prevention and treatment. This is consistent with results reported in previous studies [29–31] indicating that women with secondary or higher levels of education were more likely to use healthcare systems than those with no formal education. Plausible reasons may include the point that education enhances health literacy, equipping caregivers with knowledge about the importance of timely medical intervention [32]. Educated caregivers are more aware of the symptoms of serious illnesses like malaria and the potential risks of delayed treatment [33]. Additionally, education often correlates with improved socioeconomic status and access to resources, such as healthcare services and transportation, which further facilitates prompt care-seeking behavior [34]. This finding underscores the importance of maternal knowledge and awareness in health-seeking behavior. Educated caregivers might have better understanding and recognition of malaria symptoms and the urgency required in seeking treatment, emphasizing the need for creating educational interventions aimed at caregivers with no formal education.

Household size was another significant factor. An increase in the number of household members was associated with an increase in the likelihood of prompt malaria care-seeking. Larger households may benefit from shared responsibilities, allowing caregivers to seek timely care for their children. This finding suggests that community and family support systems are vital in facilitating prompt healthcare access. This is contrary to the result in the studies done in Ethiopia and Rwanda [31,35] which highlighted that households with many family members were less likely to seek healthcare for the sick child when compared to those having fewer household members. This could be due to the high workload, responsibilities due to large family size and financial constraints which prevented them from visiting appropriate healthcare facilities for their illnesses. This finding underscores the importance of social and familial support in health behaviors. Public health strategies should harness the strengths of larger households, such as shared responsibilities and collective knowledge, to promote timely healthcare-seeking. Nevertheless, the conflicting findings highlight the need for further research to better understand the factors influencing healthcare-seeking behavior in different family sizes. Investigating the underlying reasons for these discrepancies, including potential variations in cultural, socioeconomic, and systemic factors, will provide more nuanced insights. This additional research can help identify specific barriers and facilitators to healthcare access, enabling the development of targeted interventions and policies that address the unique needs of diverse populations.

Unexpectedly, while the use of bed nets and access to health insurance suggested a 3% and 8% increase in healthcare-seeking behavior, respectively, these factors were not statistically significant in our study. Additionally, variables such as the child’s age, parity, place of residence, marital status, wealth index, sex of the household head, and the distance to the nearest healthcare facility did not show significant associations in our context, contrary to findings in other settings. Potential reasons for these discrepancies could include regional differences in healthcare infrastructure, socioeconomic conditions, or cultural practices that influence health-seeking behaviors. The implications of these findings underscore the need for localized research to understand the unique determinants of healthcare utilization in different settings, which could inform more effective and context-specific health policies and interventions [9,11,15,36,37].

Together, these findings indicate the need for tailored policy, research and practice to address regional differences in malaria care-seeking behaviors among caregivers of under five children in Tanzania. The key findings from the analysis reveal several significant insights with important implications for policy, research, and practice. Specifically, the study highlights that female children and caregivers aged 25-34 and 35 + are associated with higher rates of prompt care-seeking, while education level and household size also influence healthcare behaviors. These findings suggest that policies should prioritize educational initiatives for caregivers and consider age-specific health interventions. Additionally, despite some factors like bed net use and health insurance showing potential associations with care-seeking, they were not statistically significant, indicating a need for more nuanced research into the effectiveness of these interventions in different contexts. For practice, healthcare providers in Tanzania should tailor their approaches to account for demographic and socioeconomic factors that influence care-seeking behavior, ensuring that interventions are relevant and accessible to all segments of the population. Further research is needed to explore these relationships in greater depth and to identify other potential barriers or facilitators to healthcare access, which can inform more targeted and effective health policies and programs.

### Strengths and limitations

To the best of our knowledge, this is the first nationally representative study in Tanzania utilizing the TDHS 2022 data to identify the determinants of prompt care seeking behavior for caregivers and their under-five children with fever. Additionally, the high response rate of over 95% indicates that the survey successfully reached and received responses from a large majority of the intended participants. This suggests strong engagement and completeness in the data collection process, enhancing the reliability and validity of the study’ findings. However, several limitations need to be acknowledged in the interpretation of the findings. First, this study used a cross-sectional design, and therefore, causality could not be determined. Moreover, the analysis was based solely on caregivers’ responses two weeks preceding the survey; therefore, recall and social desirability bias may be present. We also may have overlooked a substantial number of unreported health-seeking behaviors because the study was limited to a two-week period, whereas malaria incidence in Tanzania can be seasonal. The issue of self-medication and the use of traditional medicine were not included in the dataset, which means we missed the opportunity to explore these potentially significant and interesting variables. Future studies can investigate these missing elements to offer a more detailed understanding of prompt care-seeking behavior among caregivers and their children under five with fever.

## Conclusions

The study’s findings indicate that only 43.2%of caregivers sought healthcare for their febrile children within 24 hours of symptom onset, revealing a significant delay in accessing prompt malaria treatment. Key determinants influencing timely healthcare-seeking included the caregiver’s age, education level, socioeconomic status, and household size. This delay is particularly troubling given the high prevalence of malaria and the urgent need for timely intervention to mitigate related morbidity and mortality. We recommend educational programs to challenge gender biases and support young caregivers, promoting better health-seeking behavior and malaria awareness. We also recommend shared responsibilities in larger households to foster prompt health seeking.
